# Association of remnant cholesterol with nonalcoholic fatty liver disease: a general population-based study

**DOI:** 10.1186/s12944-021-01573-y

**Published:** 2021-10-17

**Authors:** Yang Zou, Jianyun Lan, Yanjia Zhong, Shuo Yang, Huimin Zhang, Guobo Xie

**Affiliations:** 1 From the Jiangxi Cardiovascular Research Institute, Jiangxi Provincial People’s Hospital Affiliated to Nanchang University, Nanchang, 330006 China; 2 Department of Cardiology, Jiangxi Provincial People’s Hospital Affiliated to Nanchang University, Nanchang, People’s Republic of China 330006; 3 Department of Endocrinology, Jiangxi Provincial People’s Hospital Affiliated to Nanchang University, Nanchang, People’s Republic of China 330006; 4 Department of Cardiology, Dean County People’s Hospital, Jiujiang, People’s Republic of China 330400

**Keywords:** Remnant cholesterol, General population, Nonalcoholic fatty liver disease, Lipid parameters

## Abstract

**Background:**

Remnant cholesterol (RC) mediates the progression of coronary artery disease, diabetic complications, hypertension, and chronic kidney disease. Limited information is available on the association of RC with nonalcoholic fatty liver disease (NAFLD). This study aimed to explore whether RC can be used to independently evaluate the risk of NAFLD in the general population and to analyze the predictive value of RC for NAFLD.

**Methods:**

The study included 14,251 subjects enrolled in a health screening program. NAFLD was diagnosed by ultrasound, and the association of RC with NAFLD was assessed using the receiver operating characteristic (ROC) curve and logistic regression equation.

**Results:**

Subjects with elevated RC had a significantly higher risk of developing NAFLD after fully adjusting for potential confounding factors (OR 1.77 per SD increase, 95% CI 1.64–1.91, *P* trend< 0.001). There were significant differences in this association among sex, BMI and age stratification. Compared with men, women were facing a higher risk of RC-related NAFLD. Compared with people with normal BMI, overweight and obesity, the risk of RC-related NAFLD was higher in thin people. In different age stratifications, when RC increased, young people had a higher risk of developing NAFLD than other age groups. Additionally, ROC analysis results showed that among all lipid parameters, the AUC of RC was the largest (women: 0.81; men: 0.74), and the best threshold for predicting NAFLD was 0.54 in women and 0.63 in men.

**Conclusions:**

The results obtained from this study indicate that (1) in the general population, RC is independently associated with NAFLD but not with other risk factors. (2) Compared with traditional lipid parameters, RC has a better predictive ability for NAFLD in men.

**Supplementary Information:**

The online version contains supplementary material available at 10.1186/s12944-021-01573-y.

## Background

In recent decades, the incidence of nonalcoholic fatty liver disease (NAFLD) has increased significantly globally with the prevalence of obesity and increased consumption of refined carbohydrates and saturated fats [1, 2]. A recent meta-analysis by Dr. Younossi pointed out that approximately 1/4 of adults worldwide suffer from NAFLD, among which Asia, North America and the Middle East are the most affected by NAFLD [3]. NAFLD not only brings a serious burden of liver-related diseases but also affects multiple organ systems in the whole body outside the liver, including the cardio-cerebrovascular system, musculoskeletal system, endocrine system, respiratory system and kidney organs [4–6]. Considering the prevalence of NAFLD and its many adverse health consequences, it would be helpful to reduce the disease burden of NAFLD if individuals vulnerable to NAFLD could be identified early on by simple noninvasive indicators.

Dyslipidemia is a recognized pathogenic factor of NAFLD and has been confirmed in several epidemiological and genetic studies [7, 8]. Dyslipidemia in NAFLD is similar to obesity and metabolic syndrome and is characterized by increased levels of triglyceride (TG) and decreased concentrations of high-density lipoprotein cholesterol (HDL-C) [7, 9, 10, 11]. Changes in lipid profiles can be used to predict the occurrence and progression of NAFLD. Some recent evidence suggests that remnant cholesterol (RC) rich in TG lipoprotein mediates the progression of coronary artery disease, diabetic complications, hypertension and chronic kidney disease [12–16]. However, current information on the association of RC with NAFLD is limited. The purpose of this study was to explore whether RC can be used to independently assess the risk of NAFLD in the general population and to analyze its predictive value for NAFLD.

## Methods

### Study design and data sources

NAGALA (NAfld in Gifu Area, Longitudinal Analysis) is a longitudinal survey launched in 1994 to assess risk factors for common chronic diseases in the general population. The NAGALA project continuously includes the general population who take part in a comprehensive health examination at Murakami Memorial Hospital. The details of the study design have been described elsewhere [17], in which the available public research data have been uploaded to the DRYAD database by Okamura et al. [18]. According to the DRYAD database terms of service, researchers can use the database’s exposed data to perform secondary analyses under new study assumptions. As the authorization of the ethics committee of Murakami Memorial Hospital was obtained in a previous study [17], this study does not need to be submitted for ethical approval again.

This study was a post hoc analysis of the NAGALA to investigate the association of RC with NAFLD. According to the current research purpose, the researchers extracted the available data from 2004 to 2015 in the NAGALA project (*n* = 20,944) and finally included 14,251 subjects who met the requirements according to the following exclusion criteria: (i) Missing baseline information of subjects (*n* = 863); (ii) Subjects who drank heavily (men ≥210 g/w or women ≥140 g/w; *n* = 739) [19]; (iii) Subjects were taking medication orally when baseline information was collected (*n* = 2321); (iv) Subjects with alcoholic hepatitis or viral hepatitis or diabetes or impaired fasting glucose (*n* = 1547); and (v) Subjects not enrolled in the study for unknown reasons (*n* = 10).

### Data collection and measurement

As described in a previous survey report [17], the subjects’ demographic data (age and sex), physical exercise behavior (habit of exercise), anthropometric parameters [height, weight, body mass index (BMI), waist circumference (WC) and arterial blood pressure], history of chronic diseases, medication history, and smoking and drinking status were collected and recorded by trained medical staff through standardized questionnaires. Regarding smoking status, subjects were divided into three categories according to their smoking history: non-smokers, past smokers and current smokers. Drinking status was divided into three categories: no or small drinking (< 40 g/w), light drinking (40–139 g/w), and moderate drinking (140–209 g/w), based on weekly consumption in the past month. In addition, subjects were classified as thin (< 18.5), normal BMI (≥18.5, < 25), overweight (≥25, < 30), or obese (≥30) based on a BMI cut-off point recommended by the World Health Organization for Asian populations [20]. Venous blood was collected after overnight fasting, and the levels of fasting plasma glucose (FPG), alanine aminotransferase (ALT), total cholesterol (TC), gamma-glutamyl transferase (GGT), TG, hemoglobin A1c (HbA1c), HDL-C and aspartate aminotransferase (AST) were measured using an automatic biochemistry analyzer. The low-density lipoprotein cholesterol (LDL-C) concentration was calculated using the modified Friedewald equation: LDL-C (mg/dl) = non-high-density lipoprotein cholesterol (Non-HDL-C) × 90% - TG × 10% [21]; non-HDL-C was calculated as TC minus HDL-C [21]; RC was calculated as non-HDL-C minus LDL-C [22].

### Definition of NAFLD

Abdominal ultrasound was used to diagnose NAFLD. The abdominal ultrasound procedure was performed by trained technicians, and then the gastroenterologist examined the ultrasound image without referring to the subject’s other personal data. The diagnosis of NAFLD was based on the four criteria of vascular blurring, deep attenuation, hepatorenal echo contrast and liver brightness [23].

### Statistical analysis

In this study, the statistical software Empower Stats (version 2.0) and R language (version 3.4.3) were used for data analysis, and a *P* value less than 0.05 (bilateral) was the significance criterion.

First, the quintile of RC was calculated by a quantile function, the subjects were equally divided into five groups, and the differences among each group were compared by chi-square test, nonparametric test or one-way ANOVA. The distribution pattern of continuous variables was evaluated by a QQ plot and reported as the mean (standard deviation: SD) or median (interquartile range). Categorical variables were reported as percentages.

Univariate analyses were used to initially assess the association between baseline variables and NAFLD. Then, the independent association of RC with NAFLD was evaluated by a multivariable logistic regression model, the odds ratio (OR) and 95% confidence interval (CI) of NAFLD were calculated, and the unadjusted and multivariable adjusted model analysis results were listed according to the recommendations of Strengthening the Reporting of Observational Studies in Epidemiology statement [24]. Model 1 makes preliminary adjustments to age, sex and BMI, and this model was regarded as a fine-tuning model, which can be used as a reference for further model adjustments. Model 2 regards the covariates that change at least 10% of the initial regression coefficient of the matching risk of RC-related NAFLD as confounding factors and adjusts them [25]. Model 3 was considered to be a fully adjusted model in which all non-collinear variables were adjusted (Supplementary Table 1) [26]. Additionally, the researchers used hierarchical logistic regression models to examine the association of RC with NAFLD for different ages, BMI, habit of exercise, and sex and used likelihood ratio tests to examine differences among different subgroups to determine whether there was an interaction.

Finally, to test the predictive value of lipid parameters for NAFLD, receiver operating characteristic (ROC) curves were plotted, the area under the curve (AUC) corresponding to each lipid parameter and its best threshold were calculated, and the DeLong test was used to check whether RC was significantly different from other lipid parameters.

## Results

### Baseline characteristics of subjects

Overall, 14,251 subjects met the inclusion and exclusion criteria for this study. Among these subjects, the prevalence of NAFLD was 17.59%. The baseline characteristics of the RC quintile groups by sex are shown in Table 1, and there were significant differences between groups of almost all baseline covariates (*P* < 0.05). Subjects in the highest RC group (Q5) had higher ages, LDL-C, AST, ALT, weight, height, GGT, WC, TC, BMI, TG, FPG, HbA1c, non-HDL-C and systolic/diastolic blood pressure (S/DBP) and more drinkers than other groups (Q1-Q4). Subjects with the highest RC (Q5) had lower HDL-C values than subjects with lower RC (Q1-Q4). In addition, the prevalence of NAFLD increased rapidly among the RC quintiles, and there was a significant difference, among which the prevalence rate of males was higher than that of females in all groups.

**Table 1:** Baseline characteristics of five groups

	RC quintile					*P*-value
	Q1(0.17–0.41)	Q2(0.41–0.49)	Q3(0.49–0.58)	Q4(0.58–0.71)	Q5(0.71–2.72)	
Women						
No. of subjects	2104	1699	1386	1019	632	
Age, years	38.00 (35.00–43.00)	41.00 (36.00–48.00)	45.00 (39.00–51.00)	48.00 (41.00–54.00)	51.00 (44.00–56.00)	< 0.001
BMI, kg/m^2^	19.87 (2.14)	20.57 (2.58)	21.28 (2.81)	22.16 (3.30)	23.54 (3.41)	< 0.001
WC, cm	68.98 (6.29)	70.42 (7.41)	72.28 (7.91)	74.39 (8.79)	78.28 (8.93)	< 0.001
Weight, kg	50.54 (6.34)	51.72 (7.22)	53.19 (7.80)	54.79 (8.77)	57.53 (9.31)	< 0.001
Height, cm	159.40 (5.26)	158.50 (5.39)	158.03 (5.36)	157.18 (5.06)	156.25 (5.37)	< 0.001
ALT, U/L	13.00 (10.00–16.00)	13.00 (10.00–16.00)	14.00 (11.00–17.00)	15.00 (12.00–19.00)	16.00 (13.00–22.00)	< 0.001
AST, U/L	15.00 (13.00–18.00)	16.00 (13.00–19.00)	16.00 (13.00–19.00)	17.00 (14.00–20.00)	18.00 (15.00–21.00)	< 0.001
GGT, U/L	11.00 (9.00–13.00)	11.00 (9.00–14.00)	12.00 (9.00–15.00)	12.00 (10.00–16.00)	14.00 (11.00–19.00)	< 0.001
HDL-C, mmol/L	1.74 (0.38)	1.70 (0.37)	1.64 (0.36)	1.54 (0.35)	1.34 (0.32)	< 0.001
TC, mmol/L	4.33 (0.53)	4.94 (0.54)	5.37 (0.60)	5.76 (0.66)	6.29 (0.89)	< 0.001
Non-HDL-C, mmol/L	2.59 (0.39)	3.24 (0.36)	3.73 (0.42)	4.22 (0.50)	4.95 (0.79)	< 0.001
LDL-C, mmol/L	2.24 (0.36)	2.80 (0.35)	3.20 (0.41)	3.58 (0.49)	4.11 (0.75)	< 0.001
TG, mmol/L	0.36 (0.28–0.45)	0.52 (0.43–0.62)	0.68 (0.56–0.81)	0.91 (0.76–1.08)	1.38 (1.14–1.74)	< 0.001
RC, mmol/L	0.34 (0.04)	0.45 (0.02)	0.53 (0.03)	0.63 (0.04)	0.84 (0.15)	< 0.001
FPG, mmol/L	4.88 (0.35)	4.93 (0.38)	5.03 (0.39)	5.09 (0.40)	5.21 (0.39)	< 0.001
HbA1c, %	5.11 (0.29)	5.15 (0.31)	5.21 (0.31)	5.28 (0.33)	5.36 (0.34)	< 0.001
SBP, mmHg	105.26 (11.92)	107.36 (13.10)	109.90 (14.03)	114.00 (15.06)	118.84 (16.91)	< 0.001
DBP, mmHg	64.66 (8.41)	66.44 (8.99)	68.19 (9.48)	70.68 (10.11)	73.87 (11.30)	< 0.001
Habit of exercise	297 (14.12%)	271 (15.95%)	231 (16.67%)	163 (16.00%)	117 (18.51%)	0.065
Drinking status						0.023
Non or small	1975 (93.87%)	1586 (93.35%)	1309 (94.44%)	971 (95.29%)	610 (96.52%)	
Light	129 (6.13%)	113 (6.65%)	77 (5.56%)	48 (4.71%)	22 (3.48%)	
Smoking status						< 0.001
Non	1848 (87.83%)	1512 (88.99%)	1246 (89.90%)	892 (87.54%)	538 (85.13%)	
Past	150 (7.13%)	102 (6.00%)	64 (4.62%)	59 (5.79%)	31 (4.91%)	
Current	106 (5.04%)	85 (5.00%)	76 (5.48%)	68 (6.67%)	63 (9.97%)	
NAFLD	22 (1.05%)	49 (2.88%)	94 (6.78%)	133 (13.05%)	180 (28.48%)	< 0.001
Men						
No. of subjects	746	1151	1464	1831	2219	
Age, years	38.00 (35.00–45.00)	40.00 (35.00–48.00)	42.00 (36.00–50.00)	44.00 (37.00–51.00)	44.00 (39.00–51.00)	< 0.001
BMI, kg/m^2^	21.17 (2.50)	21.74 (2.55)	22.45 (2.64)	23.29 (2.77)	24.52 (3.03)	< 0.001
WC, cm	74.82 (6.73)	76.58 (6.88)	78.85 (7.07)	81.33 (7.39)	84.35 (7.53)	< 0.001
Weight, kg	62.71 (8.75)	63.85 (8.70)	65.71 (9.02)	67.75 (9.53)	71.28 (10.40)	< 0.001
Height, cm	172.00 (6.04)	171.29 (6.02)	170.98 (6.03)	170.44 (5.94)	170.37 (5.98)	< 0.001
ALT, U/L	16.00 (13.00–20.75)	17.00 (14.00–22.00)	19.00 (15.00–25.00)	21.00 (16.00–28.00)	25.00 (19.00–35.00)	< 0.001
AST, U/L	17.00 (14.00–20.00)	18.00 (14.00–21.00)	18.00 (14.00–21.00)	18.00 (15.00–22.00)	20.00 (16.00–25.00)	< 0.001
GGT,U/L	15.00 (12.00–20.00)	16.00 (13.00–20.00)	17.00 (14.00–24.00)	19.00 (15.00–27.00)	24.00 (18.00–36.00)	< 0.001
HDL-C, mmol/L	1.52 (0.38)	1.46 (0.35)	1.36 (0.33)	1.26 (0.28)	1.11 (0.25)	< 0.001
TC, mmol/L	4.10 (0.54)	4.59 (0.52)	4.95 (0.58)	5.30 (0.62)	5.83 (0.80)	< 0.001
Non-HDL-C, mmol/L	2.58 (0.37)	3.13 (0.36)	3.59 (0.42	4.05 (0.51)	4.72 (0.74)	< 0.001
LDL-C, mmol/L	2.23 (0.35)	2.68 (0.35)	3.06 (0.41)	3.41 (0.50	3.82 (0.71)	< 0.001
TG, mmol/L	0.42 (0.33–0.50)	0.58 (0.47–0.69)	0.75 (0.63–0.87)	1.02 (0.86–1.16)	1.68 (1.39–2.16)	< 0.001
RC, mmol/L	0.35 (0.04)	0.45 (0.02)	0.53 (0.03)	0.64 (0.04)	0.90 (0.20)	< 0.001
FPG, mmol/L	5.16 (0.38)	5.22 (0.35)	5.27 (0.36)	5.32 (0.36)	5.39 (0.36)	< 0.001
HbA1c, %	5.10 (0.29)	5.13 (0.31)	5.14 (0.30	5.17 (0.32)	5.24 (0.33)	< 0.001
SBP, mmHg	113.98 (12.50)	115.24 (13.07)	116.56 (13.31)	118.75 (13.94)	121.90 (14.47)	< 0.001
DBP, mmHg	70.52 (9.33)	71.94 (9.28)	73.26 (9.18)	74.99 (9.71)	77.26 (9.98)	< 0.001
Habit of exercise	202 (27.08%)	243 (21.11%)	306 (20.90%)	310 (16.93%)	330 (14.87%)	< 0.001
Drinking status						0.175
Non or small	532 (71.31%)	821 (71.33%)	1029 (70.29%)	1345 (73.46%)	1627 (73.32%)	
Light	149 (19.97%)	218 (18.94%)	304 (20.77%)	320 (17.48%)	378 (17.03%)	
Moderate	65 (8.71%)	112 (9.73%)	131 (8.95%)	166 (9.07%)	214 (9.64%)	
Smoking status						< 0.001
Non	344 (46.11%)	468 (40.66%)	546 (37.30%)	616 (33.64%)	736 (33.17%)	
Past	186 (24.93%)	340 (29.54%)	419 (28.62%)	581 (31.73%)	627 (28.26%)	
Current	216 (28.95%)	343 (29.80%)	499 (34.08%)	634 (34.63%)	856 (38.58%)	
NAFLD	55 (7.37%)	108 (9.38%)	251 (17.14%)	524 (28.62%)	1091 (49.17%)	< 0.001

Values were expressed as mean (standard deviation) or medians (quartile interval) or n (%). *Abbreviations*: *BMI* body mass index, *WC* Waist circumference, *ALT* alanine aminotransferase, *AST* aspartate aminotransferase, *GGT* gamma-glutamyl transferase, *HDL-C* high-density lipoprotein cholesterol, *TC* total cholesterol, *Non-HDL-C* Non-high-density lipoprotein-cholesterol, *LDL-C* Low density lipoprotein cholesterol, *TG* triglyceride, *RC* remnant cholesterol, *HbA1c* hemoglobin A1c, *FPG* fasting plasma glucose, *SBP* systolic blood pressure, *DBP* Diastolic blood pressure, *NAFLD* Nonalcoholic fatty liver disease

### Association of RC with NAFLD

First, the association between baseline variables and NAFLD was assessed by univariate analysis. It is evident from Table 2 that RC was most strongly associated with NAFLD risk. For the sake of description, the researchers performed a multivariate analysis after converting the RC to Z score. Table 3 shows the relationship between RC and the risk of NAFLD. In the unadjusted model, there was a significant positive correlation between RC and the risk of NAFLD, and the risk of NAFLD corresponding to the quintile of RC gradually increased (OR 2.83 per SD increase, 95% CI 2.70–2.98, *P* trend< 0.001). In the age-, sex- and BMI-adjusted model (Model 1), the positive correlation between RC and NAFLD was slightly weaker than that in the unadjusted model, and the trend of the positive correlation remained unchanged. After further adjustment for sex, BMI, TC, FPG and SBP (Model 2), the degree of positive correlation between them changed slightly, and the risk of NAFLD increased by 88% for each increase in 1 SD by RC (OR 1.88 per SD increase, 95% CI 1.75–2.01). Finally, after adjusting all non-collinear covariables, RC and NAFLD maintained a positive correlation, the degree of correlation between them decreased slightly, and the linear trend of RC from the lowest to the highest quintile was significant (OR 1.77 per SD increase, 95% CI 1.64–1.91, *P* trend< 0.001).

**Table 2:** The results of univariate analysis

	Statistics	OR (95% CI)	*P*-value
Sex			< 0.0001
Women	6840 (48.00%)	Ref	
Men	7411 (52.00%)	5.02 (4.51, 5.58)	
Age, years	43.53 ± 8.89	1.02 (1.01, 1.02)	< 0.0001
BMI, kg/m^2^	22.06 ± 3.14	1.65 (1.61, 1.68)	< 0.0001
WC, cm	76.19 ± 9.10	1.20 (1.19, 1.21)	< 0.0001
Weight, kg	60.26 ± 11.61	1.13 (1.12, 1.14)	< 0.0001
Height, cm	164.80 ± 8.48	1.06 (1.05, 1.06)	< 0.0001
Habit of exercise	2470 (17.33%)	0.82 (0.72, 0.92)	0.0008
ALT, U/L	19.76 ± 14.47	1.10 (1.10, 1.11)	< 0.0001
AST, U/L	18.23 ± 8.67	1.09 (1.08, 1.10)	< 0.0001
GGT, U/L	19.13 ± 16.13	1.04 (1.04, 1.04)	< 0.0001
HDL-C, mmol/L	1.46 ± 0.40	0.05 (0.05, 0.06)	< 0.0001
TC, mmol/L	5.12 ± 0.87	1.65 (1.57, 1.73)	< 0.0001
Non-HDL-C, mmol/L	3.66 ± 0.91	2.42 (2.30, 2.55)	< 0.0001
LDL-C, mmol/L	3.09 ± 0.75	2.40 (2.26, 2.54)	< 0.0001
TG, mmol/L	0.89 ± 0.63	4.65 (4.30, 5.03)	< 0.0001
RC, mmol/L	0.57 ± 0.21	153.60 (120.88, 195.19)	< 0.0001
FPG, mmol/L	5.15 ± 0.41	6.91 (6.13, 7.78)	< 0.0001
HbA1c, %	5.18 ± 0.32	4.42 (3.84, 5.08)	< 0.0001
SBP, mmHg	113.93 ± 14.82	1.05 (1.05, 1.06)	< 0.0001
DBP, mmHg	71.12 ± 10.38	1.08 (1.07, 1.08)	< 0.0001
Drinking status			
Non or small	11,805 (82.84%)	Ref	
Light	1758 (12.34%)	0.90 (0.79, 1.04)	0.1442
Moderate	688 (4.83%)	1.12 (0.92, 1.36)	0.2731
Smoking status			
Non	8746 (61.37%)	Ref	
Past	2559 (17.96%)	2.12 (1.91, 2.37)	< 0.0001
Current	2946 (20.67%)	1.93 (1.73, 2.14)	< 0.0001

Values were expressed as mean (standard deviation) or medians (quartile interval) or n (%)

Note: “Statistics” refers to the statistical description of the baseline variable

*Abbreviations*: *OR* Odds ratios, *CI* confidence interval; other abbreviations as in Table 1

**Table 3:** Logistic regression analyses for the association between RC and NAFLD in different models

	OR (95% CI)						*P*-trend
	Multivariable Analysis (OR per SD increase)	Q1	Q2	Q3	Q4	Q5	
Unadjusted Model	2.83 (2.70, 2.98)	Ref	2.10 (1.59, 2.77)	4.96 (3.85, 6.39)	10.79 (8.46, 13.75)	28.97 (22.83, 36.76)	< 0.001
Model 1	1.79 (1.69, 1.89)	Ref	1.27 (0.95, 1.71)	2.14 (1.63, 2.81)	3.28 (2.52, 4.27)	6.01 (4.63, 7.81)	< 0.001
Model 2	1.88 (1.75, 2.01)	Ref	1.36 (1.01, 1.84)	2.32 (1.75, 3.08)	3.77 (2.84, 5.01)	7.30 (5.42, 9.85)	< 0.001
Model 3	1.77 (1.64, 1.91)	Ref	1.41 (1.03, 1.93)	2.29 (1.70, 3.09)	3.55 (2.63, 4.80)	6.43 (4.68, 8.85)	< 0.001

*Abbreviations*: *RC* remnant cholesterol, *OR* Odds ratios, *CI* confidence interval

Model 1 adjusted for sex, age and BMI

Model 2 adjusted for sex, BMI, TC, FPG and SBP

Model 3 adjusted for sex, age, BMI, ALT, AST, height, habit of exercise, GGT, TC, FPG, HbA1c, SBP, drinking status and smoking status

### Subgroup analysis

The researchers also analyzed the association of RC with NAFLD after stratification by sex, BMI, age and habit of exercise and examined the differences among different subgroups by the likelihood ratio test (Table 4). These results suggest that there were significant differences in NAFLD risk among RC patients stratified by age, sex and BMI. Compared with men, women were facing a higher risk of RC-related NAFLD (OR = 2.35 per SD increase for women, OR = 1.79 per SD increase for men; *P*-interaction = 0.0001). Compared with people with normal BMI, overweight and obesity, the risk of RC-related NAFLD was higher in thin people (OR = 3.49 per SD increase for thin people, OR = 2.19 per SD increase for people with normal BMI, OR = 1.80 per SD increase for overweight people, OR = 1.78 per SD increase for obese people; *P*-interaction = 0.0097). In different age stratifications, when RC increased, young people had a higher risk of developing NAFLD than other age groups.

**Table 4:** Stratified associations between RC and NAFLD by age, sex, BMI and habit of exercise

Subgroup	No. of participants	unadjusted OR (95%CI)	adjusted 0R (95%CI)	*P-*interaction
Age (years)				0.0004
18–29	401	7.22 (3.71, 14.04)	3.79 (1.83, 7.82)	
30–44	7901	3.40 (3.16, 3.66)	2.07 (1.89, 2.27)	
44–59	5314	2.35 (2.18, 2.53)	1.68 (1.53, 1.84)	
≥ 60	635	1.81 (1.47, 2.24)	1.66 (1.34, 2.05)	
Sex				0.0001
Women	6840	3.47 (3.10, 3.88)	2.35 (2.05, 2.70)	
Men	7402	2.24 (2.12, 2.37)	1.79 (1.67, 1.93)	
BMI (kg/m^2^)				0.0097
< 18.5	1545	3.92 (1.48, 10.39)	3.49 (1.31, 9.27)	
≥ 18.5, < 25	10,442	2.43 (2.28, 2.58)	2.19 (2.02, 2.36)	
≥ 25, < 30	2012	1.86 (1.69, 2.05)	1.80 (1.62, 2.01)	
≥ 30	252	1.78 (1.25, 2.75)	1.78 (1.19, 2.65)	
Habit of exercise				0.3073
Yes	2470	2.48 (2.20, 2.79)	1.76 (1.54, 2.01)	
No	11,781	2.48 (2.20, 2.79)	1.90 (1.76, 2.05)	

*Abbreviations*: *OR* Odds ratios, *CI* confidence interval; other abbreviations as in Table 1, adjusted for sex, age, BMI, ALT, AST, height, habit of exercise, GGT, TC, FPG, HbA1c, SBP, drinking status and smoking status

### ROC analysis

**Fig. 1:**
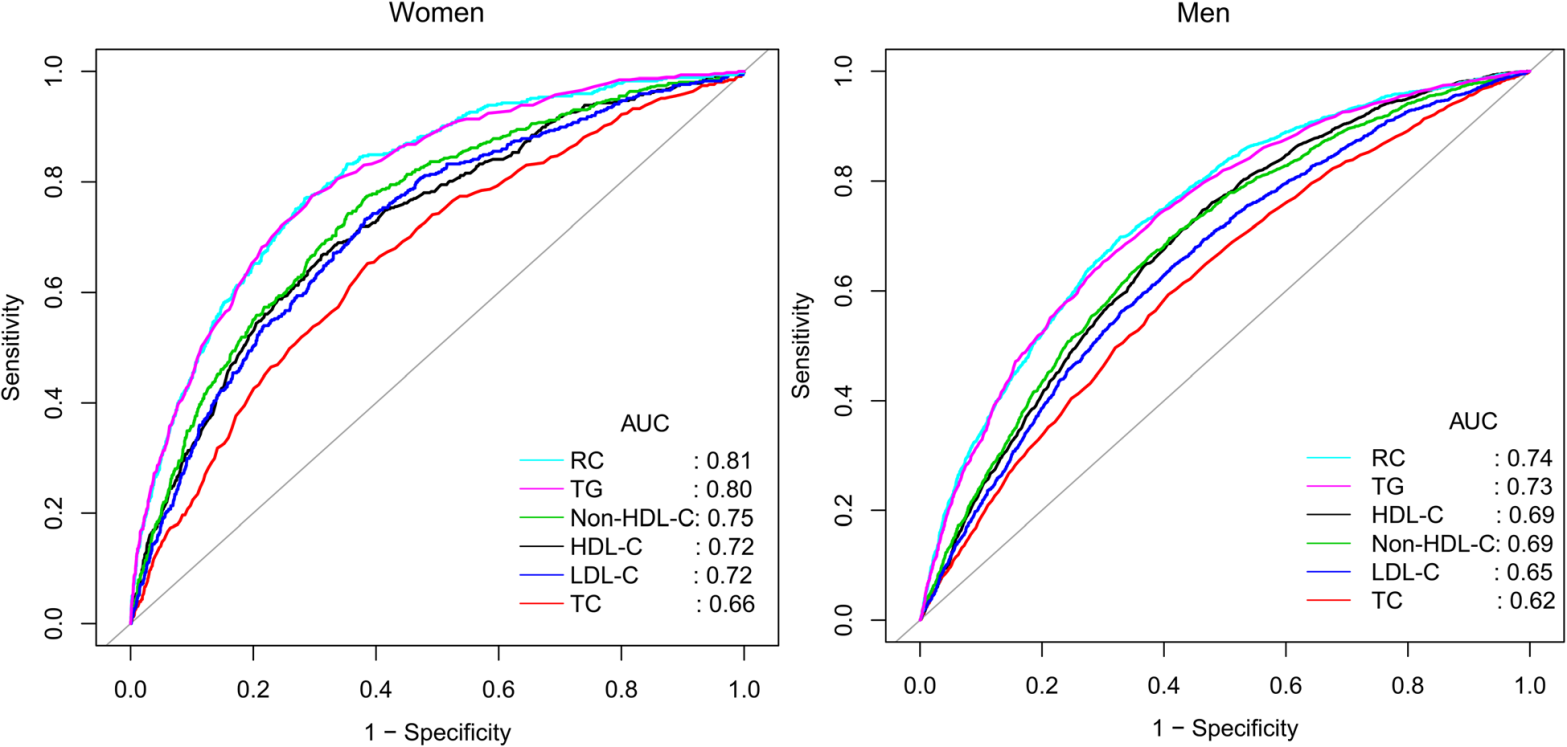
ROC curve analysis of NAFLD-related lipid parameters in men and women. ROC: receiver operating characteristic; NAFLD: nonalcoholic fatty liver disease; HDL-C: high-density lipoprotein cholesterol; TC: total cholesterol; TG: triglyceride; Non-HDL-C: non-high-density lipoprotein cholesterol; LDL-C: Low-density lipoprotein cholesterol; RC: remnant cholesterol

To evaluate the predictive value of non-HDL-C, LDL-C, TG, HDL-C, TC and RC in NAFLD, ROC analysis was performed to calculate the AUC of each lipid parameter in different sexes. As shown in Table 5 (Fig. 1), the AUC of RC was the highest among all lipid parameters, which was 0.74 in men and 0.81 in women. It is worth mentioning that in men, the predictive value of RC for NAFLD was significantly better than that of other lipid parameters. Among women, the predictive value of RC for NAFLD was better than that of LDL-C, non-HDL-C, TC and HDL-C, but there was no significant difference between AUC and TG.

**Table 5:** Areas under the receiver operating characteristic curves for each lipid parameters in identifying nonalcoholic fatty liver disease

	AUC	DeLong test *P*-value	95%CI low	95%CI upp	Best threshold	Specificity	Sensitivity
Women							
HDL-C, mmol/L	0.72	< 0.0001	0.70	0.75	1.46	0.6888	0.6674
TC, mmol/L	0.66	< 0.0001	0.64	0.69	5.24	0.6136	0.6527
Non-HDL-C, mmol/L	0.75	< 0.0001	0.72	0.77	3.55	0.6152	0.7741
LDL-C, mmol/L	0.72	< 0.0001	0.70	0.74	3.05	0.6180	0.7322
RC, mmol/L	0.81		0.79	0.83	0.54	0.7163	0.7699
TG, mmol/L	0.80	0.7978	0.79	0.82	0.72	0.7047	0.7741
Men							
HDL-C, mmol/L	0.69	< 0.0001	0.67	0.70	1.27	0.5367	0.7467
TC, mmol/L	0.62	< 0.0001	0.61	0.63	5.21	0.5927	0.5934
Non-HDL-C, mmol/L	0.69	< 0.0001	0.67	0.70	3.85	0.5909	0.6964
LDL-C, mmol/L	0.65	< 0.0001	0.64	0.67	3.39	0.6702	0.5623
RC, mmol/L	0.74		0.73	0.75	0.63	0.6722	0.6979
TG, mmol/L	0.73	0.0418	0.72	0.75	1.07	0.6997	0.6530

*Abbreviations*: *AUC* area under the curve, *CI* confidence interval; other abbreviations as in Table 1

The DeLong test was used to compare the AUC of RC with other lipid parameters in men and women

## Discussion

In this cross-sectional study based on the general population, it was observed that there was an independent positive correlation between RC and NAFLD. After fully adjusting for other covariates, the risk of NAFLD increased by 88% for every 1 SD increase in RC. This association also existed in different subgroups, and there were significant differences. In addition, the predictive value of RC for NAFLD was better than that of other lipid parameters in men.

Dyslipidemia is a recognized pathogenic factor of NAFLD and has been confirmed in several epidemiological and genetic studies [7, 8]. Among them, the increase in intrahepatic TG accompanied by insulin resistance is an important characteristic [27, 28]. However, in recent years, some studies have proposed other explanations. In an experimental study conducted by the Yamaguchi team, they pointed out that TG does not seem to be lipotoxic and is more like an inert lipid [29]. The pathogenesis of NAFLD may be closely related to the accumulation of free cholesterol in the liver and the imbalance of cholesterol homeostasis in the liver [30–32]. According to the description of Nuño-Lámbarri et al., an imbalance in liver cholesterol will further aggravate the accumulation of free cholesterol in the liver [33]. In addition to these findings, Ducheix et al. pointed out that excessive accumulation of cholesterol in cells will activate the liver X receptor, which further induces or aggravates steatosis of the liver [34]. Therefore, atherogenic dyslipidemia in peripheral blood may indicate cholesterol accumulation in hepatocytes and a higher risk of NAFLD.

### Comparisons with other studies and what does the current work add to the existing knowledge

RC is cholesterol-rich in TG lipoprotein, which consists of chylomicron remnants, intermediate-density lipoprotein and very-low-density lipoprotein [35]. It can integrate a variety of atherosclerotic effects, including monocyte activation, upregulation of proinflammatory cytokines and increased production of thrombogenic factors [35, 36]. Adverse cardiovascular events related to RC have also been observed in many clinical studies. High levels of RC are reported to be associated with an increased risk of coronary artery disease, diabetic complications, hypertension and chronic kidney disease [12–16]. Recently, there have been several reports on the relationship between RC and NAFLD. In 2018, Pastori et al. found for the first time that higher levels of RC were independently positively correlated with NAFLD in patients with cardiac metabolic diseases [37], and Chin et al. also found a similar association in adolescents [38]. This conclusion was validated in this study, and the findings of this study support the role of RC as an independent risk factor for NAFLD in the general population. On this basis, further analysis also found that the predictive value of RC for NAFLD was significantly better than that of other lipid parameters in men. Additionally, in a recent study by Campanella et al., they investigated 237 patients with metabolic syndrome who participated in a randomized controlled trial and found that there was a correlation between RC and the severity of NAFLD [39]. This finding suggests that RC has potential application value in monitoring the occurrence and progression of NAFLD.

Another important finding of the current study is that there are significant differences in the corresponding NAFLD risk of RC in different age, sex and BMI stratifications. Compared with men, women have a higher risk of developing NAFLD with higher RC levels. However, the prevalence of NAFLD in men was still higher than that in women in the group with higher levels of RC in this study. Therefore, there may be some special reasons for the higher risk of NAFLD associated with RC in women. To explain this phenomenon, the researchers summarized the baseline characteristics according to sex in the group with the highest RC value (Supplementary Table 2). The results showed that in the group with the highest RC levels, female NAFLD patients had more significant dyslipidemia than male NAFLD patients in this study. This finding suggests that the disorder of blood lipid metabolism may be an important reason for the sex dependence of RC-related NAFLD risk, and the higher adiponectin concentration in women may be an important factor [40]. In addition, female NAFLD patients in the group with the highest level of RC in this study were significantly older than male NAFLD patients. Therefore, the increased risk of RC-related NAFLD in women may also be related to the lack of adequate estrogen in women of higher age [41]. Among people with different BMI phenotypes, it is particularly noteworthy that thin people face a higher risk of RC-related NAFLD. Some recent studies have shown that non-obese people seem to be more likely to have metabolic disorders [42–44]. More surprisingly, in a recent global survey of non-obese or lean NAFLD, it was revealed that approximately 40% of the global NAFLD population is currently classified as non-obese and nearly one-fifth as lean [45]. In different age stratifications, when RC increased, young people had a higher risk of developing NAFLD than other age groups. With the obesity epidemic, young people may already be overweight or obese in childhood [1, 46]. With the accelerated development of a wide range of new media and unhealthy living habits, young people experience all kinds of metabolic problems prematurely [47, 48]. Additionally, although the interaction between the habit of exercise and RC is not significant, it can be observed from the stratified analysis that people who maintain exercise habits have a lower risk of developing NAFLD. At present, the treatment of NAFLD mainly focuses on improving lifestyle and increasing exercise [2]. Perhaps with further research on the association of RC with NAFLD, the treatment plan for the regulation of RC may become a new research direction.

### Study strength and limitation

The advantages of this study are as follows: (i) The sample size of this study is large, it has been strictly statistically adjusted, and the final conclusion can be considered to be relatively objective. (ii) This study found that RC is more valuable in predicting NAFLD than traditional lipid parameters in men. (iii) RC is simple to calculate and easy to obtain. It is especially suitable for chronic disease risk assessment and epidemiological investigation in the general population.

A few limitations should also be highlighted: (i) Due to the cross-sectional design of this study, the causal association between RC and NAFLD needs to be explored in further longitudinal studies. (ii) In this study, NAFLD was evaluated and diagnosed according to ultrasound. Compared with liver biopsy, abdominal ultrasound may not be very sensitive to some mild hepatic steatosis [49]. (iii) Although the covariables have been statistically adjusted by various methods in this study, it is undeniable that there are still some unmeasured or undiscovered influencing factors that are not included, which may lead to some residual confounding [50].

## Conclusion

In conclusion, this study demonstrates that RC is an independent risk factor for NAFLD in the general population and is a better predictor of NAFLD risk than conventional lipid parameters in men. RC is a novel and clinically effective marker that is simple to calculate and easy to obtain and can be used to identify people at high risk of NAFLD in the early stage.

## Supplementary Information

**Additional file 1: Supplementary Table 1.** Collinearity diagnostics steps. **Supplementary Table 2.** Baseline characteristics of NAFLD patients in Q5 group of RC.

## Data Availability

The datasets that support the conclusions of this article can be found in the Dryad repository.

## Abbreviations

RC: Remnant cholesterol
NAFLD: nonalcoholic fatty liver disease
ROC: Receiver operating characteristic
TG: Triglyceride
LDL-C: Low density lipoprotein cholesterol
HDL-C: High density lipoprotein cholesterol
WC: Waist circumference
BMI: Body mass index
FPG: Fasting plasma glucose
ALT: Alanine aminotransferase
HbA1c: Haemoglobin A1c
GGT: Gamma-glutamyl transferase
AST: Aspartate aminotransferase
Non-HDL-C: Non-high-density lipoprotein-cholesterol
SD: Standard deviation
OR: Odds ratio
CI: Confidence interval
AUC: Area under the curve
S/DBP: Systolic/diastolic blood pressure
TC: Total cholesterol
NAGALA: NAfld in Gifu Area, Longitudinal Analysis

## References

[1] Milić S, Lulić D, Štimac D (2014). Non-alcoholic fatty liver disease and obesity: biochemical, metabolic and clinical presentations. World J Gastroenterol.

[2] Hernandez-Rodas MC, Valenzuela R, Videla LA (2015). Relevant aspects of nutritional and dietary interventions in non-alcoholic fatty liver disease. Int J Mol Sci.

[3] Younossi ZM, Koenig AB, Abdelatif D, Fazel Y, Henry L, Wymer M (2016). Global epidemiology of nonalcoholic fatty liver disease-Meta-analytic assessment of prevalence, incidence, and outcomes. Hepatology.

[4] Ballestri S, Mantovani A, Nascimbeni F, Lugari S, Lonardo A (2019). Extra-hepatic manifestations and complications of nonalcoholic fatty liver disease. Future Med Chem.

[5] Byrne CD, Targher G (2015). NAFLD: a multisystem disease. J Hepatol.

[6] Adams LA, Anstee QM, Tilg H, Targher G (2017). Non-alcoholic fatty liver disease and its relationship with cardiovascular disease and other extrahepatic diseases. Gut.

[7] Katsiki N, Mikhailidis DP, Mantzoros CS (2016). Non-alcoholic fatty liver disease and dyslipidemia: an update. Metabolism.

[8] Gaggini M, Morelli M, Buzzigoli E, DeFronzo RA, Bugianesi E, Gastaldelli A (2013). Non-alcoholic fatty liver disease (NAFLD) and its connection with insulin resistance, dyslipidemia, atherosclerosis and coronary heart disease. Nutrients.

[9] Zhang QQ, Lu LG (2015). Nonalcoholic fatty liver disease: dyslipidemia, risk for cardiovascular complications, and treatment strategy. J Clin Transl Hepatol.

[10] Bloomgarden ZT (2004). Dyslipidemia and the metabolic syndrome. Diabetes Care.

[11] Grundy SM (2016). Metabolic syndrome update. Trends Cardiovasc Med.

[12] Joshi PH, Khokhar AA, Massaro JM, Lirette ST, Griswold ME, Martin SS, Blaha MJ, Kulkarni KR, Correa A, D'Agostino RB, Jones SR, Toth PP, the Lipoprotein Investigators Collaborative (LIC) Study Group (2016). Remnant lipoprotein cholesterol and incident coronary heart disease: the Jackson heart and Framingham offspring cohort studies. J Am Heart Assoc.

[13] Li K, Fan F, Zheng B, Jia J, Liu B, Liu J, Chen C, Zhou J, Zhang Y, Huo Y (2021). Associations between remnant lipoprotein cholesterol and central systolic blood pressure in a Chinese community-based population: a cross-sectional study. Lipids Health Dis.

[14] Hong LF, Yan XN, Lu ZH, Fan Y, Ye F, Wu Q, Luo SH, Yang B, Li JJ (2017). Predictive value of non-fasting remnant cholesterol for short-term outcome of diabetics with new-onset stable coronary artery disease. Lipids Health Dis.

[15] Yan P, Xu Y, Miao Y, Bai X, Wu Y, Tang Q, et al. Association of remnant cholesterol with chronic kidney disease in middle-aged and elderly Chinese: a population-based study. Acta Diabetol. 2021. 10.1007/s00592-021-01765-z. 10.1007/s00592-021-01765-z 34181081

[16] Cao YX, Zhang HW, Jin JL, Liu HH, Zhang Y, Gao Y, Guo YL, Wu NQ, Hua Q, Li YF, Li XL, Xu RX, Cui CJ, Liu G, Dong Q, Sun J, Zhu CG, Li JJ (2020). The longitudinal association of remnant cholesterol with cardiovascular outcomes in patients with diabetes and pre-diabetes. Cardiovasc Diabetol.

[17] Okamura T, Hashimoto Y, Hamaguchi M, Obora A, Kojima T, Fukui M (2019). Ectopic fat obesity presents the greatest risk for incident type 2 diabetes: a population-based longitudinal study. Int J Obes.

[18] Okamura T, et al. Data from: ectopic fat obesity presents the greatest risk for incident type 2 diabetes: a population-based longitudinal study. Dryad, Dataset. 2019. 10.5061/dryad.8q0p192. 10.1038/s41366-018-0076-3 29717276

[19] Choi JH, Sohn W, Cho YK (2020). The effect of moderate alcohol drinking in nonalcoholic fatty liver disease. Clin Mol Hepatol.

[20] Expert WHO (2004). Consultation. Appropriate body-mass index for Asian populations and its implications for policy and intervention strategies. Lancet.

[21] Chen Y, Zhang X, Pan B, Jin X, Yao H, Chen B, Zou Y, Ge J, Chen H (2010). A modified formula for calculating low-density lipoprotein cholesterol values. Lipids Health Dis.

[22] Nordestgaard BG, Varbo A (2014). Triglycerides and cardiovascular disease. Lancet.

[23] Hamaguchi M, Kojima T, Itoh Y, Harano Y, Fujii K, Nakajima T, Kato T, Takeda N, Okuda J, Ida K, Kawahito Y, Yoshikawa T, Okanoue T (2007). The severity of ultrasonographic findings in nonalcoholic fatty liver disease reflects the metabolic syndrome and visceral fat accumulation. Am J Gastroenterol.

[24] Fitchett EJA, Seale AC, Vergnano S, Sharland M, Heath PT, Saha SK, Agarwal R, Ayede AI, Bhutta ZA, Black R, Bojang K, Campbell H, Cousens S, Darmstadt GL, Madhi SA, Meulen AS, Modi N, Patterson J, Qazi S, Schrag SJ, Stoll BJ, Wall SN, Wammanda RD, Lawn JE, SPRING (Strengthening Publications Reporting Infection in Newborns Globally) Group (2016). Strengthening the reporting of observational studies in epidemiology for newborn infection (STROBE-NI): an extension of the STROBE statement for neonatal infection research. Lancet Infect Dis.

[25] Vandenbroucke JP, von Elm E, Altman DG, Gøtzsche PC, Mulrow CD, Pocock SJ, Poole C, Schlesselman JJ, Egger M, STROBE Initiative (2014). Strengthening the reporting of observational studies in epidemiology (STROBE): explanation and elaboration. Int J Surg.

[26] Kim JH (2019). Multicollinearity and misleading statistical results. Korean J Anesthesiol.

[27] Birkenfeld AL, Shulman GI (2014). Nonalcoholic fatty liver disease, hepatic insulin resistance, and type 2 diabetes. Hepatology.

[28] Zou Y, Sheng G, Yu M, Xie G (2020). The association between triglycerides and ectopic fat obesity: an inverted U-shaped curve. PLoS One.

[29] Yamaguchi K, Yang L, McCall S, Huang J, Yu XX, Pandey SK, Bhanot S, Monia BP, Li YX, Diehl AM (2007). Inhibiting triglyceride synthesis improves hepatic steatosis but exacerbatesliver damage and fibrosis in obese mice with nonalcoholic steatohepatitis. Hepatology.

[30] Fon Tacer K, Rozman D (2011). Nonalcoholic fatty liver disease: focus on lipoprotein and lipid deregulation. J Lipids.

[31] Min H-K, Kapoor A, Fuchs M, Mirshahi F, Zhou H, Maher J, Kellum J, Warnick R, Contos MJ, Sanyal AJ (2012). Increased hepatic synthesis and dysregulation of cholesterol metabolism is associated with the severity of nonalcoholic fatty liver disease. Cell Metab.

[32] Van Rooyen DM, Larter CZ, Haigh WG, Yeh MM, Ioannou G, Kuver R (2011). Hepatic free cholesterol accumulates in obese, diabetic mice and causes nonalcoholic steatohepatitis. Gastroenterology.

[33] Nuño-Lámbarri N, Domínguez-Pérez M, Baulies-Domenech A, Monte MJ, Marin JJ, Rosales-Cruz P (2016). Liver cholesterol overload aggravates obstructive cholestasis by inducing oxidative stress and premature death in mice. Oxidative Med Cell Longev.

[34] Ducheix S, Montagner A, Theodorou V, Ferrier L, Guillou H (2013). The liver X receptor: a master regulator of the gut-liver axis and a target for non alcoholic fatty liver disease. Biochem Pharmacol.

[35] Twickler TB, Dallinga-Thie GM, Cohn JS, Chapman MJ (2004). Elevated remnant-like particle cholesterol concentration: a characteristic feature of the atherogenic lipoprotein phenotype. Circulation.

[36] Varbo A, Benn M, Tybjærg-Hansen A, Nordestgaard BG (2013). Elevated remnant cholesterol causes both low-grade inflammation and ischemic heart disease, whereas elevated low-density lipoprotein cholesterol causes ischemic heart disease without inflammation. Circulation.

[37] Pastori D, Baratta F, Novo M, Cocomello N, Violi F, Angelico F, del Ben M (2018). Remnant lipoprotein cholesterol and cardiovascular and cerebrovascular events in patients with non-alcoholic fatty liver disease. J Clin Med.

[38] Chin J, Mori TA, Adams LA, Beilin LJ, Huang RC, Olynyk JK, Ayonrinde OT (2020). Association between remnant lipoprotein cholesterol levels and non-alcoholic fatty liver disease in adolescents. JHEP Rep.

[39] Campanella A, Iacovazzi PA, Misciagna G, Bonfiglio C, Mirizzi A, Franco I, Bianco A, Sorino P, Caruso MG, Cisternino AM, Buongiorno C, Liuzzi R, Osella AR (2020). The effect of three Mediterranean diets on remnant cholesterol and non-alcoholic fatty liver disease: a secondary analysis. Nutrients.

[40] Cnop M, Havel PJ, Utzschneider KM, Carr DB, Sinha MK, Boyko EJ, Retzlaff BM, Knopp RH, Brunzell JD, Kahn SE (2003). Relationship of adiponectin to body fat distribution, insulin sensitivity and plasma lipoproteins: evidence for independent roles of age and sex. Diabetologia.

[41] Nasr A, Breckwoldt M (1998). Estrogen replacement therapy and cardiovascular protection: lipid mechanisms are the tip of an iceberg. Gynecol Endocrinol.

[42] Kwon YM, Oh SW, Hwang SS, Lee C, Kwon H, Chung GE (2012). Association of nonalcoholic fatty liver disease with components of metabolic syndrome according to body mass index in Korean adults. Am J Gastroenterol.

[43] Zou Y, Zhong L, Hu C, Sheng G (2020). Association between the alanine aminotransferase/aspartate aminotransferase ratio and new-onset non-alcoholic fatty liver disease in a nonobese Chinese population: a population-based longitudinal study. Lipids Health Dis.

[44] Sheng G, Xie Q, Wang R, Hu C, Zhong M, Zou Y (2021). Waist-to-height ratio and non-alcoholic fatty liver disease in adults. BMC Gastroenterol.

[45] Ye Q, Zou B, Yeo YH, Li J, Huang DQ, Wu Y, Yang H, Liu C, Kam LY, Tan XXE, Chien N, Trinh S, Henry L, Stave CD, Hosaka T, Cheung RC, Nguyen MH (2020). Global prevalence, incidence, and outcomes of non-obese or lean non-alcoholic fatty liver disease: a systematic review and meta-analysis. Lancet Gastroenterol Hepatol.

[46] Cuthbertson DJ, Brown E, Koskinen J, Magnussen CG, Hutri-Kähönen N, Sabin M, Tossavainen P, Jokinen E, Laitinen T, Viikari J, Raitakari OT, Juonala M (2019). Longitudinal analysis of risk of non-alcoholic fatty liver disease in adulthood. Liver Int.

[47] American College of Obstetricians and Gynecologists’ Committee on Adolescent Health Care (2016). Committee Opinion No. 653: Concerns Regarding Social Media and Health Issues in Adolescents and Young Adults. Obstet Gynecol.

[48] McMahon DM, Burch JB, Youngstedt SD, Wirth MD, Hardin JW, Hurley TG (2019). Relationships between chronotype, social jetlag, sleep, obesity and blood pressure in healthy young adults. Chronobiol Int.

[49] Ferraioli G, Soares Monteiro LB (2019). Ultrasound-based techniques for the diagnosis of liver steatosis. World J Gastroenterol.

[50] Black N (1996). Why we need observational studies to evaluate the effectiveness of health care. BMJ.

