# Supplementary material for: Association of remnant cholesterol with nonalcoholic fatty liver disease: a general population-based study
Source: Lipids Health Dis. 2021 Oct 17;20:139. doi: 10.1186/s12944-021-01573-y (PMC8520640; doi:10.1186/s12944-021-01573-y)
Supplement: Supplementary file 1 — Additional file 1: Supplementary Table 1. Collinearity diagnostics steps. Supplementary Table 2. Baseline characteristics of NAFLD patients in Q5 group of RC. [file 12944_2021_1573_MOESM1_ESM.docx]

Supplementary Table 1: Collinearity diagnostics steps.

|  | Step 1 | Step 2 | Step 3 | Step 4 | Step 5 | Step 6 | Step 7 | Step 8 |
| --- | --- | --- | --- | --- | --- | --- | --- | --- |
| RC, mmol/L | Inf | Inf | Inf | 48.7 | 48.6 | 2.3 | 2.3 | 2.3 |
| Sex | 3.3 | 3.3 | 3.3 | 3.3 | 3.3 | 3.2 | 3.2 | 3.2 |
| Age, years | 1.4 | 1.4 | 1.4 | 1.4 | 1.4 | 1.4 | 1.3 | 1.3 |
| BMI, kg/m^2^ | 95.9 | 95.9 | 95.9 | 95.9 | 5 | 5 | 1.6 | 1.6 |
| WC, cm | 5.9 | 5.9 | 5.9 | 5.9 | 5.9 | 5.9 | NA | NA |
| ALT, U/L | 4.1 | 4.1 | 4.1 | 4.1 | 4.1 | 4.1 | 4.1 | 4.1 |
| AST, U/L | 3.3 | 3.3 | 3.3 | 3.3 | 3.3 | 3.3 | 3.3 | 3.3 |
| Weight, kg | 168.5 | 168.6 | 168.6 | 168.6 | NA | NA | NA | NA |
| Height, cm | 51.9 | 51.9 | 51.9 | 51.9 | 2.9 | 2.9 | 2.4 | 2.4 |
| Habit of exercise | 1 | 1 | 1 | 1 | 1 | 1 | 1 | 1 |
| GGT, U/L | 1.5 | 1.5 | 1.5 | 1.5 | 1.5 | 1.4 | 1.4 | 1.4 |
| HDL-C, mmol/L | Inf | NA | NA | NA | NA | NA | NA | NA |
| TC, mmol/L | Inf | 7.4 | 7.4 | 7.4 | 7.4 | 1.8 | 1.8 | 1.8 |
| Non-HDL-C, mmol/L | Inf | Inf | NA | NA | NA | NA | NA | NA |
| LDL-C, mmol/L | Inf | Inf | Inf | NA | NA | NA | NA | NA |
| TG, mmol/L | Inf | Inf | Inf | 30.6 | 30.5 | NA | NA | NA |
| FPG, mmol/L | 1.5 | 1.5 | 1.5 | 1.5 | 1.5 | 1.5 | 1.5 | 1.5 |
| HbA1c, % | 1.2 | 1.2 | 1.2 | 1.2 | 1.2 | 1.2 | 1.2 | 1.2 |
| Drinking status | 1.2 | 1.2 | 1.2 | 1.2 | 1.2 | 1.2 | 1.2 | 1.2 |
| Smoking status | 1.4 | 1.4 | 1.4 | 1.4 | 1.4 | 1.4 | 1.4 | 1.4 |
| SBP, mmHg | 5.5 | 5.5 | 5.5 | 5.5 | 5.5 | 5.5 | 5.5 | 1.4 |
| DBP, mmHg | 5.6 | 5.6 | 5.6 | 5.6 | 5.6 | 5.6 | 5.6 | NA |

Abbreviations: Inf: infinity; VIF: Variance inflation factor; Other abbreviations as in Table ​1.

Note: VIF = 1/(1-R^2^).

Supplementary Table 2: Baseline characteristics of NAFLD patients in Q5 group of RC.

|  | Women | Men | *P*-value |
| --- | --- | --- | --- |
| No. of subjects | 180 | 1091 |  |
| Age, years | 49.40 (44.00-55.00) | 43.00 (38.00-50.00) | <0.001 |
| BMI, kg/m^2^ | 25.96 (3.11) | 25.89 (3.01) | 0.769 |
| WC, cm | 84.38 (9.01) | 87.53 (7.18) | <0.001 |
| ALT, U/L | 21.00 (16.00-28.00) | 31.00 (23.00-44.50) | <0.001 |
| AST, U/L | 19.00 (17.00-22.00) | 22.00 (18.00-27.00) | <0.001 |
| GGT, U/L | 16.00 (13.00-22.00) | 28.00 (20.00-40.00) | <0.001 |
| Weight, kg | 63.65 (9.62) | 75.35 (10.61) | <0.001 |
| Height, cm | 156.47 (5.30) | 170.44 (5.88) | <0.001 |
| HDL-C, mmol/L | 1.23 (0.26) | 1.07 (0.22) | <0.001 |
| TC, mmol/L | 6.16 (0.82) | 5.81 (0.77) | <0.001 |
| Non-HDL-C, mmol/L | 4.93 (0.75) | 4.75 (0.73) | 0.004 |
| LDL-C, mmol/L | 4.04 (0.69) | 3.81 (0.68) | <0.001 |
| TG, mmol/L | 1.55 (1.26-1.93) | 1.82 (1.48-2.33) | <0.001 |
| RC, mmol/L | 0.83 (0.76-0.96) | 0.89 (0.79-1.02) | <0.001 |
| FPG, mmol/L | 5.33 (0.38) | 5.44 (0.35) | <0.001 |
| HbA1c, % | 5.47 (0.32) | 5.30 (0.33) | <0.001 |
| SBP, mmHg | 123.27 (17.23) | 125.29 (14.68) | 0.06 |
| DBP, mmHg | 76.55 (10.44) | 79.55 (10.11) | <0.001 |
| Habit of exercise | 28 (15.56%) | 150 (13.75%) | 0.518 |
| Drinking status |  |  | <0.001 |
| Non or small | 177 (98.33%) | 860 (78.83%) |  |
| Light | 3 (1.67%) | 151 (13.84%) |  |
| Moderate | 0 (0.00%) | 80 (7.33%) |  |
| Smoking status |  |  | <0.001 |
| Non | 151 (83.89%) | 388 (35.56%) |  |
| Past | 9 (5.00%) | 310 (28.41%) |  |
| Current | 20 (11.11%) | 393 (36.02%) |  |

Abbreviations as in Table ​1.
